# Applications of Machine Learning in Real-Life Digital Health Interventions: Review of the Literature

**DOI:** 10.2196/12286

**Published:** 2019-04-05

**Authors:** Andreas K Triantafyllidis, Athanasios Tsanas

**Affiliations:** 1 Information Technologies Institute Centre for Research and Technology Hellas Thessaloniki Greece; 2 Lab of Computing, Medical Informatics and Biomedical Imaging Technologies School of Medicine Aristotle University of Thessaloniki Thessaloniki Greece; 3 Usher Institute of Population Health Sciences and Informatics Medical School University of Edinburgh Edinburgh United Kingdom; 4 Mathematical Institute University of Oxford Oxford United Kingdom

**Keywords:** machine learning, data mining, artificial intelligence, digital health, review, telemedicine

## Abstract

**Background:**

Machine learning has attracted considerable research interest toward developing smart digital health interventions. These interventions have the potential to revolutionize health care and lead to substantial outcomes for patients and medical professionals.

**Objective:**

Our objective was to review the literature on applications of machine learning in real-life digital health interventions, aiming to improve the understanding of researchers, clinicians, engineers, and policy makers in developing robust and impactful data-driven interventions in the health care domain.

**Methods:**

We searched the PubMed and Scopus bibliographic databases with terms related to machine learning, to identify real-life studies of digital health interventions incorporating machine learning algorithms. We grouped those interventions according to their target (ie, target condition), study design, number of enrolled participants, follow-up duration, primary outcome and whether this had been statistically significant, machine learning algorithms used in the intervention, and outcome of the algorithms (eg, prediction).

**Results:**

Our literature search identified 8 interventions incorporating machine learning in a real-life research setting, of which 3 (37%) were evaluated in a randomized controlled trial and 5 (63%) in a pilot or experimental single-group study. The interventions targeted depression prediction and management, speech recognition for people with speech disabilities, self-efficacy for weight loss, detection of changes in biopsychosocial condition of patients with multiple morbidity, stress management, treatment of phantom limb pain, smoking cessation, and personalized nutrition based on glycemic response. The average number of enrolled participants in the studies was 71 (range 8-214), and the average follow-up study duration was 69 days (range 3-180). Of the 8 interventions, 6 (75%) showed statistical significance (at the *P*=.05 level) in health outcomes.

**Conclusions:**

This review found that digital health interventions incorporating machine learning algorithms in real-life studies can be useful and effective. Given the low number of studies identified in this review and that they did not follow a rigorous machine learning evaluation methodology, we urge the research community to conduct further studies in intervention settings following evaluation principles and demonstrating the potential of machine learning in clinical practice.

## Introduction

### Background

Digital health interventions [1], including modalities such as telemedicine, Web-based strategies, email, mobile phones, mobile apps, text messaging, and monitoring sensors, have enormous potential to support independent living and self-management [2], and reduce health care costs [3]. They have also shown great promise in improving health [4]. With the advent of new tools and algorithms for machine learning, a new class of smart digital health interventions can be developed, which could revolutionize effective health care delivery [5].

The term *machine learning* is widely used across disciplines but has no universally accepted definition [6]. This is in part explained by the breadth of the areas it covers and because researchers from diverse disciplines have historically contributed (and still contribute) to its development. Broadly, it refers to an algorithmic framework that can provide insights into data, while facilitating inference and providing a tentative setting to determine functional relationships.

Machine learning has been applied in multiple health care domains, including diabetes [7], cancer [8], cardiology [9], and mental health [10]. Most of the developed machine learning models and tools in research settings have investigated the potential of prognosis [11], diagnosis [12], or differentiation of clinical groups (eg, a group with a pathology and a healthy control group or groups with pathologies) [13], thus demonstrating promise toward the development of computerized decision support tools [14]. The key requirements for the development of these tools are sufficiently large datasets (in terms of both number of participants and explanatory variables to explore) and accurate labels, typically provided by expert clinicians. The premise is the identification of those data structures or variables (eg, clinical, behavioral, or demographic variables) that are associated with the target outcome (eg, whether a person has cancer). In this regard, useful knowledge can be derived from the available data, which can empower patients to monitor their health status longitudinally and support health professionals in decision making with regard to management, treatment, and follow-up interventions where required.

Despite a considerably growing body of research literature in the use of machine learning in health care applications [15], it is astonishing how few of these suggestions are actually translated into clinical practice [16]. There is remarkably limited empirical evidence of the effectiveness of machine learning applications in digital health interventions. This is rather surprising, since any proposed health care solutions would reach their full potential only if they are embraced by the medical community, becoming integrated within properly designed digital health interventions and tested in real-life studies with patients and health professionals.

### Objective

Considering that machine learning models and tools have not been widely and reliably used in clinical practice, whereas the peer-reviewed literature in the field is growing exponentially, we wanted to assess the progress made in smart data-driven health interventions applied in real-life research settings—that is, the real world in which constraints in available resources or opportunities to collect reliable data may exist, as opposed to simulation or laboratory-based studies [17]. In this direction, we present a systematic literature review of digital health interventions incorporating machine learning algorithms, by identifying and mapping their features and outcomes, with the aim to improve our knowledge of the design and development of impactful intelligent interventions.

## Methods

### Inclusion and Exclusion Criteria

We sought to identify digital health nonpharmacological interventions incorporating machine learning that were assessed in pragmatic studies. In this context, the inclusion criteria for study selection were (1) the study should be conducted with patients or health professionals, or both, in a real-life setting, (2) machine learning algorithms or models were used in the digital health intervention (rather than merely reporting statistical hypothesis testing results or statistical associations), (3) quantitative outcomes of the study were presented, and (4) the article describing the study was written in English. We excluded retrospective studies, case reports, ongoing studies, surveys or reviews, laboratory or simulation studies, studies describing protocols, qualitative studies, and all studies published before 2008 from the review because we wanted to determine the status of recent research developments in the field that have been used in clinical interventional settings.

### Literature Search and Screening

We searched the PubMed and Scopus bibliographic databases for studies published after 2008 using the string “(machine learning) OR (data mining) OR (artificial intelligence) AND health” for search within the title, abstract, and keywords of the articles. We limited “Species” in PubMed to humans.

Both authors independently screened the identified articles following the literature search to minimize bias in the selection process. Any disagreements were resolved by discussion between the authors and reaching a consensus. We screened the abstracts of the candidate articles for inclusion and subsequently read the full text of the articles deemed eligible according to the inclusion criteria. Subsequently, we excluded articles not providing sufficient information about the application of machine learning or for being ineligible. We used the Effective Public Health Practice Project (EPHPP) tool to assess the methodological quality of the included studies, which has been found to be reliable [18]. The studies that focused on interventions were synthesized (AKT) according to their target (ie, target condition), study design, number of enrolled participants, follow-up duration, primary outcome and whether this was significantly positive, machine learning algorithms used in the intervention, and outcome of the algorithms (eg, prediction of a target outcome).

The systematic review was conducted following the Preferred Reporting Items for Systematic Reviews and Meta-Analyses (PRISMA) guidelines [19]. Multimedia Appendix 1 shows a completed PRISMA checklist.

## Results

### Literature Search Outcomes

**Figure 1:**
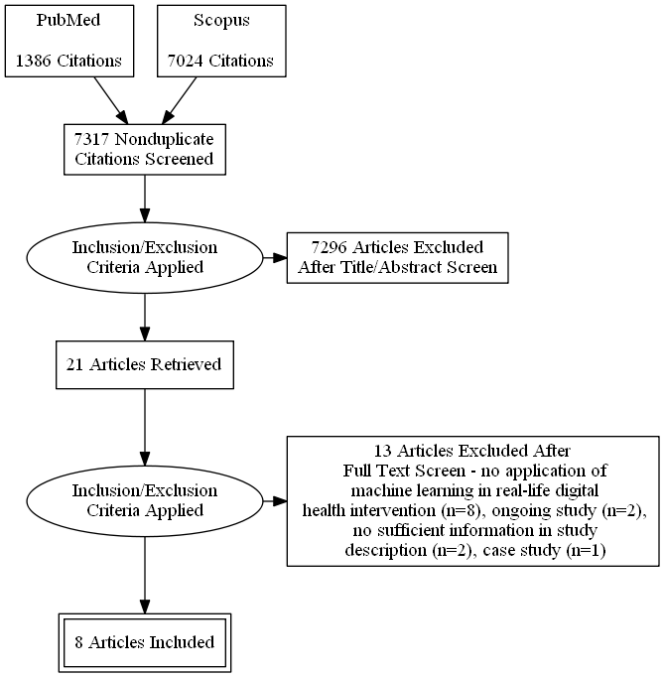
Flow diagram for study inclusion following the Preferred Reporting Items for Systematic Reviews and Meta-Analyses (PRISMA) format.

Our last search in November 2018 returned 1386 articles from the PubMed database and 7024 articles from Scopus. We imported all the retrieved records into Mendeley (version 1.19.3) bibliography management software (Mendeley Ltd) [20], which identified 1093 duplicates. We screened the abstracts of the remaining 7317 results according to our inclusion and exclusion criteria and identified 21 eligible articles. The reviewers read the full text of the 21 articles and agreed on 8 for inclusion as eligible articles. The flow diagram in Figure 1 summarizes the reasons for excluding research articles for study inclusion following the PRISMA format (Figure 1).

### Quality Assessment

On the basis of the EPHPP criteria for selection bias, design, confounders, blinding, data collection, and dropouts, we found the methodological quality to be moderate for 2 of the 8 (25%) studies [21,22] and weak for the remaining 6 (75%) studies [23-28] (Table 1). Most studies were poorly rated because of selection bias, insufficient care in controlling for confounders, and the high percentage of withdrawals or dropouts (or the absence of their description). The design of a randomized or controlled clinical trial was described in 3 (37%) studies [21,25,28], and 5 (63%) interventions were evaluated in a pilot or experimental single-group study (Multimedia Appendix 2).

**Table 1:** Quality assessment of included studies based on Effective Public Health Practice Project (EPHPP) criteria.

Study first author, year, and reference	EPHPP criteria						Global rating
	Selection bias	Study design	Confounders	Blinding	Data collection methods	Withdrawals and dropouts	
Burns, 2011 [23]	Weak	Moderate	Weak	Moderate	Strong	Strong	Weak
Hawley, 2013 [24]	Weak	Weak	Weak	Moderate	Weak	Weak	Weak
Manuvinakurike, 2014 [22]	Weak	Strong	Strong	Moderate	Moderate	Strong	Moderate
Martin, 2012 [25]	Moderate	Strong	Weak	Moderate	Strong	Weak	Weak
Morrison, 2017 [26]	Moderate	Moderate	Weak	Strong	Strong	Weak	Weak
Ortiz-Catalan, 2016 [27]	Moderate	Moderate	Weak	Moderate	Weak	Weak	Weak
Sadasivam, 2016 [21]	Weak	Strong	Strong	Strong	Moderate	Strong	Moderate
Zeevi, 2015 [28]	Weak	Strong	Weak	Moderate	Weak	Weak	Weak

### Type of Intervention and Target Population

The interventions targeted depression prediction and management [23], speech recognition for people with speech disabilities [24], self-efficacy for weight loss [22], detection of changes in biopsychosocial condition of patients with multiple morbidity [25], stress management [26], treatment of phantom limb pain [27], smoking cessation [21], and personalized nutrition based on glycemic response [28] (Multimedia Appendix 2).

Of the 8 interventions, 3 (37%) targeted patients: individuals with a diagnosis of depression [23], those with multiple morbidities such as lung disease and cardiovascular disease [25], and those with phantom limb pain [27]. One (13%) intervention targeted people with speech disabilities [24]; 4 (50%) interventions targeted individuals who had no explicit diagnosis of a disease or impairment [21,22,26,28]. All target groups comprised adults. The average number of enrolled participants in the studies was 71 (range 8-214), and the average follow-up study duration was 69 days (range 3-180).

### Applications of Machine Learning and Outcomes

Overall, 6 of the 8 (75%) real-life studies of digital health interventions aided by machine learning algorithms showed statistical significance (at the *P*=.05 level) in health outcomes. Different summary measures were used in the identified studies to assess primary outcomes, which reflects the lack of standardization both in methodology and in the metrics used in the research fields. Where possible, we aimed to use the accuracy of the algorithms used and the *P* value (eg, for showing statistical significance of outcomes in an intervention group compared with a control group) as the principal summary measures. We briefly describe all included studies below in terms of intervention purpose and content, evaluation outcomes, and implications for clinical practice.

Burns et al [23] described a multicomponent mobile-based intervention that used machine learning models to predict the mood, emotions, cognitive and motivational states, activities, and environmental and social context of patients with depression, along with feedback graphs for self-reflection on behavior and coaching provided by caregivers. The predictive models were based on phone sensor–derived variables (eg, global positioning system, ambient light, phone calls), and regression along with decision trees was used. The accuracy of the models was promising for location prediction (60%-91%), but prediction was very poor for emotions such as sadness. Overall, the 8 participants in the study became less likely to meet the criteria for a diagnosis of major depressive disorder (*P*=.03), and their symptoms of depression and anxiety were decreased by the end of the study (*P*<.001). Patients were also satisfied with the intervention (5.71 average rating on a scale 1 to 7), and 6 of 7 treatment completers (86%) indicated that the intervention was helpful in understanding triggers for negative moods. Despite the benefits of self-reflection on behavior through the use of a multicomponent mobile health monitoring system and the clinical improvements shown in the study, the authors reported that the clinical utility of the prediction models they used should be improved, since the prediction outcomes (eg, location and mood) were merely displayed to the users, and there were no direct interventions based on them.

Hawley et al [24] described the use of a device capable of recognizing the speech of people with dysarthria and generating voice messages. The authors used hidden Markov models to determine the proximity of a spoken word to a personalized speech model for that individual. However, only 67% recognition accuracy was achieved in this real-life observational study with 9 participants. Participants noticed that ease of communication was reduced through the device compared with their usual communication method of either speaking or speaking supported by a conventional voice-output communication aid, mainly due to the low accuracy of speech recognition. Nevertheless, feedback from participants was positive about the device’s concept, given that speech recognition was improved.

Manuvinakurike et al [22] focused on changes in self-efficacy for weight loss through the provision of personal health behavior change stories found on the internet. An algorithm based on adaptive boosting was developed to find the most relevant story based on the stage of change and the demographic characteristics of a user, along with the emotional tone and overall quality of the story (accuracy between 84% and 98% for the classification of 5 stages of change). Testing of the algorithm with 103 users revealed significantly greater increases in self-efficacy for weight loss (*P*=.02) and a statistically insignificant effect on change in decisional balance (*P*=.83). In addition, the medium used to tell the stories, being either text or an animated conversational agent, had no effect on health behavior change. The authors concluded that their approach could maximize participants’ engagement in longitudinal health behavior change interventions.

Martin et al [25] used a system in which decision trees could predict unplanned hospital visits of patients with multiple morbidities such as lung disease or cardiovascular disease. Alerts were sent to health professionals, who acted on the alerts according to agreed guidelines. The system was based on information received via patient phone calls with lay care guides. Linguistic and metalinguistic features were extracted, together with the patient’s status, to train the prediction models (positive predictive value of 70% for predicting unplanned events). A randomized controlled trial with 214 patients for 6 months (the largest trial we found in the review in terms of number of enrolled participants and duration) showed a reduction of 50% in the number of unplanned hospital events of participants in the intervention group compared with control. The most common response to an alert indicating that a patient needed attention (red alert) was to phone the patient the next day to reassess the situation and contact their general practitioner (3% of calls), suggest or plan a visit to their general practitioner (11% of calls), or call an ambulance (<0.01% of calls). In summary, the authors reported that predictive analytics on an ongoing basis could be used to signify risk of hospitalization and guide the health care system to take appropriate actions.

Morrison et al [26] used push notifications to enhance engagement of smartphone users for stress management. They used a naïve Bayes classifier to predict whether a user would respond to a notification, thereby building a personalized intelligent mechanism for notification delivery, based on the times within a day a user was more likely to view and react to the received messages. However, this exploratory study with 77 participants showed no statistically significant difference between participants receiving the messages sent “intelligently” and those receiving a message daily or occasionally within 72 hours (Cohen *d*=0.14 for intelligent vs daily group and *d*=0.5 for intelligent vs occasional group, for actions taken in response to messages). Although notification delivery based on time had no effect on the study groups (ie, response to notifications was no different), the authors concluded that frequent daily messages may not deter users from engaging with digital health interventions.

Ortiz-Catalan et al [27] applied myoelectric pattern recognition algorithms for the control of a virtual limb in patients with phantom limb pain and used gaming along with augmented and virtual reality for treatment. This single-group study with 14 participants revealed that patients’ symptoms of phantom limb pain were significantly decreased (by about 50%) at the end of the provided treatment for 6 months (*P*=.0001 for reduction in intensity and quality of pain). The authors suggested that their novel treatment could be used after failure of evidence-based treatments such as mirror therapy and before proceeding with invasive or pharmacological approaches.

Sadasivam et al [21] used a recommender system to send motivational messages to individuals, targeting smoking cessation. The system was based on Bayesian probabilistic matrix factorization to predict message rating, through the processing of data from the user’s previous ratings of messages, along with other users’ ratings. This randomized controlled trial with 120 users showed that the system was more effective at influencing people to quit smoking than were standard tailored messages (rule-based system) with proven effectiveness (*P*=.02) and resulted in a similar cessation rate. The authors concluded that their recommender system could be used instead of standard systems for influencing smoking cessation because it was more personalized (it learned and adapted to a person’s behavior) and could incorporate a considerably greater number of variables; however, larger trials would be needed to demonstrate the system’s effectiveness.

Zeevi et al [28] used gradient boosting regression to predict the postmeal glycemic response of individuals in real life, according to blood parameters, dietary habits, anthropometrics, physical activity, and gut microbiota. The results from this randomized controlled study with 24 participants showed that a personalized diet based on postmeal glycemic predictions could statistically significantly modify elevated postprandial blood glucose (*P*<.05 for predicting low levels of blood glucose [“good diet”] vs high levels of blood glucose [“bad diet”], which was comparable with diets selected by experts). The authors reported that their approach could be used in nutritional interventions for controlling or preventing disorders associated with poor glycemic control, such as obesity, diabetes, and nonalcoholic fatty liver disease. However, evaluation periods of months or even years would be needed first to clearly indicate the effectiveness of the proposed algorithm.

## Discussion

### Principal Findings

This review is, to our knowledge, the first to systematically examine the features and outcomes of digital health interventions incorporating machine learning that were implemented and assessed in real-life studies [17]. With this aim in mind, we differentiated our review from previous investigations that focused only on the broader use of artificial intelligence in medicine in the context of specific diseases [29,30], machine learning techniques [31,32], or risk prediction models, such as through mining of electronic health records [33,34], and did not consider real-life evaluation of the respective interventions. The need to demonstrate evidence of an intervention’s effectiveness in the real world has been highlighted in several other studies [35-37]. Our main finding is that most of the digital health interventions showed significantly positive health outcomes for patients or healthy individuals, which demonstrates the virtue of machine learning applications in actual clinical practice. However, given the small number of studies identified in this review and their considerable limitations highlighted above, further work is warranted to demonstrate the effectiveness of digital interventions relying on machine learning applications in real-life medical care.

Our review found 8 different cases of machine learning applications in a real-life setting: depression prediction and management, speech recognition for people with speech disabilities, self-efficacy for weight loss, detection of changes in biopsychosocial condition of patients with multiple morbidity, stress management, treatment of phantom limb pain, smoking cessation, and personalized nutrition based on glycemic response. The reviewed studies had several implications for clinical practice, such as better engagement of patients with interventions [22], the identification of risk for hospitalization [25], or the introduction of novel treatment methods [27]. Among the studies, those for speech recognition of people with speech disabilities [24] and notification delivery for stress management [26] clearly reported insignificant outcomes, whereas 6 studies showed significant outcomes, but they were of low to moderate methodological quality. Only 3 studies were in the form of a randomized controlled trial, which limited the ability to fully identify the added value of machine learning-enabled interventions compared with standard care. To this end, further rigorous studies with adequately powered samples (recruiting considerably more participants than the average number of 71 participants found in this review) are needed, which would generate the evidence base for the effectiveness of machine learning in clinical practice. To that effect, large trials and publicly accessible databases that have become available over the last few years, such as the UK BioBank and the Physionet database, are providing rich resources that could facilitate insights.

The delivery of motivational messages [21,26] or stories [22] for health behavior change and engagement seems to be an emerging area of digital health interventions incorporating machine learning. These studies also demonstrated the latest efforts to promote individuals’ personalized self-management and to put them at the center of health care [38]. Considering the effectiveness of tailored messaging in influencing health behavior change [39], further research in this area is warranted.

The surprisingly small number of identified pragmatic studies in our review might raise some concerns and indicates the substantial challenge of systematically evaluating digital health interventions that incorporate machine learning [40]. In this context, the retrospective validation of algorithms and models, given the availability of one or more datasets, constitutes only the first step in the evaluation process [28]. The second step involves the integration of the algorithms and models within a digital health tool, such as mobile phone–based tools [23], internet-based tools [14], or an aid device [24]. The third step requires the assessment of the developed tool as a digital health intervention in a real-life research setting (eg, through a randomized controlled trial), together with patients or health professionals, or both [28,41]. The final step would be the monitoring of actual uptake and use of the intervention in real-world settings and outside of a research setting [42], which is, however, rarely reported [43]. Admittedly, this process is challenging and anything but trivial. It requires a significant amount of time and resources, which might not always be available, and multidisciplinary collaboration among experts in different fields, such as engineering, computer science, behavioral science, and medicine, which might not be straightforward. However, such synergistic collaborative approaches are likely necessary in the development of evidence-based, sustainable, and impactful digital health interventions [44,45].

### Limitations

We used the term *machine learning*, along with broader terms such as *data mining* and *artificial intelligence*, for our literature search, rather than keywords for specific machine learning algorithms or domains relevant to digital health, such as *telemedicine*. This might have inadvertently omitted studies that could have contributed to the progress made in machine learning applications for digital health. We combined the aforementioned terms with the generic term *health*, aiming to conduct a broad search within the provided boundaries and to include the most pertinent articles relevant to digital health. We searched for articles in a limited number of databases (ie, PubMed and Scopus), which nevertheless are two of the most widely used databases internationally [46]. We did not hand search any studies reported in other reviews or the included studies, and we did not assess the interrater reliability. A meta-analysis was not possible due to the heterogeneity of the included studies.

### Conclusion

Our review showed that real-life digital health interventions incorporating machine learning can be useful and effective. Considering the small number of studies examined in this review and their limitations, further evidence of the clinical usefulness of machine learning in health service delivery is needed. We encourage researchers to move beyond the retrospective validation of machine learning models, by integrating their models within appropriately designed digital health tools and evaluating their tools in rigorous studies conducted in real-life settings.

## Appendix

Multimedia Appendix 1 PRISMA checklist.

Multimedia Appendix 2 Characteristics of included studies and implications for clinical practice.

## Abbreviations

EPHPP: Effective Public Health Practice Project
PRISMA: Preferred Reporting Items for Systematic Reviews and Meta-Analyses
