# Supplementary material for: Applications of Machine Learning in Real-Life Digital Health Interventions: Review of the Literature
Source: J Med Internet Res. 2019 Apr 5;21(4):e12286. doi: 10.2196/12286 (PMC6473205; doi:10.2196/12286)
Supplement: Multimedia Appendix 2 [file jmir_v21i4e12286_app2.pdf]

Characteristics of included studies and implications for clinical practice.

	Intervention target	Study design	NP[a]	Follow-up duration	Primary outcome	SO[b]	Machine learning algorithm(s) used	Machine learning algorithm outcome	Implications for clinical practice
Burns, 2011 [23]	Depression prediction and management	Single-group interventional study	8	8 weeks	Severity of depressive symptoms	Yes	Regression and decision trees	Prediction of sadness (7-point Likert scale) and location	To better understand an individual's daily behavior (eg, triggers of negative emotions)
Hawley, 2013 [24]	Speech recognition for people with speech disabilities	Observational study	9	2-4 weeks	Recognition accuracy in real settings	No	Hidden Markov models	Closeness of fit of user attempt in speaking a word to own speech recognition model	To provide a comfortable aid for the patient if achieved accuracy is good
Manuvinakurike, 2014 [22]	Self-efficacy for weight loss	2×2 between-subjects randomized pilot study	103	N/A[c]	Self-efficacy for weight loss	Yes (No for decisional balance)	Adaptive boosting	Relevance of stories to the users	To maximize participants' engagement in longitudinal health behavior change interventions
Martin, 2012 [25]	Detection of changes in biopsychosocial condition of patients with multiple morbidity (eg, lung disease and cardiovascular disease)	Randomized controlled trial	214	6 months	Unplanned hospital visits	Yes	Decision trees	Prediction of unplanned events	To signify the risk of hospitalization
Morrison, 2017 [26]	Stress management	Exploratory mixed-methods study	77	3 days on average	Patterns of use and notification response	No	Naïve Bayesian classifier	Notification response (yes/no)	To expose users to intervention content without deterring engagement
Ortiz-Catalan, 2016 [27]	Treatment of phantom limb pain	Single-group interventional study	14	6 months	Phantom limb pain	Yes	Linear discriminant analysis, multilayer perceptron (supervised artificial neural network), self-organized map (unsupervised	Prediction of individual and simultaneous movements	To be used in novel treatment methods that could be used in case evidence-based treatment fails

							artificial neural network), regulatory feedback networks		
Sadasivam, 2016 [21]	Smoking cessation	Randomized controlled trial	120	30 days	Perceived influence to quit via motivational messages	Yes	Bayesian probabilistic matrix factorization	Prediction of user rating of messages (5-point Likert scale)	To influence individuals to change bad health habits
Zeevi, 2015 [28]	Personalized nutrition based on glycemic response	Randomized controlled trial	24	2 weeks	Postmeal glycemic response	Yes	Gradient boosting regression	Prediction of postmeal glycemic response	To prevent or control disorders such as diabetes and obesity

[a]NP: number of enrolled participants.

[b]SO: significantly positive outcome reported.

[c]N/A: not applicable.
